# Research note: Effects of dietary *Abelmoschus manihot* L. flower powder supplementation on growth performance, relative organ weight, and liver status in broiler chickens

**DOI:** 10.1016/j.psj.2025.105853

**Published:** 2025-09-16

**Authors:** Gyu Lim Yeom, Ha Neul Lee, Yeong Bin Kim, Ju Yeong Park, Geun Yong Park, Ji Won Shin, Yang-il Choi, Jungseok Choi, Jong Hyuk Kim

**Affiliations:** a Department of Animal Science*,* Chungbuk National University, Cheongju 28644, Republic of Korea; b Research Center, Orge CO., LTD., Jecheon 27157, Republic of Korea

**Keywords:** *Abelmoschus manihot* L. flower, Antioxidant capacity, Broiler chicken, Growth performance, Liver

## Abstract

The objective of this experiment was to assess the impact of dietary *Abelmoschus manihot* L. flower powder (**AMP**) supplementation on growth performance, relative organ weight, and liver status in broiler chickens. A total of 144 1-d-old mixed-sex Arbor Acres broiler chicks were randomly allotted to 1 of 3 dietary treatments with 6 replicates per treatment in a completely randomized design. Each replicate contained 8 birds. The corn-soybean meal-based basal diet was prepared without AMP supplementation. Two additional experimental diets were formulated by incorporating AMP into the basal diet at levels of 0.5 or 1.0 %. The diets were provided *ad libitum* for 35 d. The results indicated that dietary AMP supplementation has no positive effects on growth performance. However, there was a trend toward reduced (linear, *P* = 0.054) relative liver weight as AMP inclusion increased in diets. Moreover, birds fed diets containing 1.0 % AMP had less relative spleen weight than those fed diets containing 0 and 0.5 % AMP, and there was a decrease (linear, *P* < 0.05) in relative spleen weight as inclusion levels of AMP in diets increased. Birds fed diets containing 0.5 and 1.0 % AMP had less (*P* < 0.05) fatty liver scores than those fed the basal diet. Increasing inclusion levels of AMP in diets also decreased (linear and quadratic, *P* < 0.05) fatty liver score in broiler chickens. Feeding diets supplemented with 1.0 % AMP tended to increase (*P* = 0.085) total antioxidant capacity (**TAC**) than those fed basal diet, and TAC increased (linear, *P* < 0.05) by increasing inclusion levels of AMP in diets. Collectively, dietary AMP supplementation improves hepatic function and increases antioxidant capacity in broiler chickens, with no detrimental effects observed in this experiment. These findings support that AMP may be considered a promising feed additive in broiler diets to improve hepatic health of broiler chickens.

## Introduction

Among poultry, broiler chickens have been especially valued due to their rapid growth, efficient feed efficiency, and short production cycles, which collectively provide notable advantages compared to other livestock. However, accelerated growth frequently coincides with weakened immune responses, thereby raising the risk of pathogen colonization and inflammatory processes that further damage intestinal structure and disrupt barrier integrity in broiler chickens. To address these issues, antibiotic growth promoters were traditionally incorporated into broiler diets to support intestinal function, bolster resilience to stress, and inhibit disease (Windisch et al., 2008). However, their ban has led to a search for growth promotors such as phytogenic feed additives (**PFAs**). Most PFAs display a range of beneficial properties such as antioxidant, anti-inflammatory, antimicrobial, immunomodulatory, and metabolism-modulating activities that underpin enhanced feed efficiency, nutrition utilization, intestinal health, animal well-being, and overall performance (Flees et al., 2021).

*Abelmoschus manihot* L. is a member of the *Malvaceae* family and is commonly known as sunset marshmallow, sunset hibiscus, or hibiscus manihot and widely found throughout tropical regions. It is widely found throughout tropical regions. Traditionally, *Abelmoschus manihot* L. has been utilized in humans via both topical and oral administration to alleviate swelling, assist in detoxification, promote blood circulation and hemostasis, and support urination. This plant contains an array of bioactive constituents, such as saponins, alkaloids, steroids, flavonoids, and triterpenoids, each possessing anti-inflammatory, antioxidant, and antimicrobial properties (Kwon et al., 2022). In poultry, the roots and leaves of *Abelmoschus manihot* L. contain abundant mucilage and nutrients, which may play a role in supporting general health. In contrast, the flowers contain higher concentrations of bioactive compounds, such as flavonoids, and relatively lower fiber concentrations, which are appropriate for use as a functional feed additive without adversely affecting nutrient absorption. Nevertheless, research on the effects of dietary supplementation of *Abelmoschus manihot* L. flower powder (AMP) in broiler chickens remains limited. Therefore, this study was conducted to investigate the impact of dietary AMP supplementation on growth performance, relative organ weight, and liver status in broiler chickens.

## Materials and methods

### Sample preparation

*Abelmoschus manihot* L. flowers were freshly collected from a local farm (Kounblack, Goesan-gun, Republic of Korea). The flowers were oven-dried at 40°C for 48 h and then ground to obtain a particle size of less than 1 mm prior to refrigeration. The composition of AMP was determined using standard analytical procedures. The crude protein (CP), amino acid, calcium, and phosphorus concentrations were assessed based on the analyzed nutrient compositions of AMP. The nutrient composition of AMP has been indicated in Table 1.

**Table 1:** Composition and nutrient contents of the experimental diets and *Abelmoschus manihot* L. flower powder (as-fed basis).

Items	Grower (0 - 21 d), %	Finisher (22 - 35 d), %
Ingredients		
Corn	56.1	60.7
Soybean meal, 46 % CP	30.3	27.7
Corn gluten meal	6.42	3.46
Soybean oil	2.24	3.29
Salt	0.300	0.300
MCP	1.68	1.24
Limestone	1.23	0.900
98.5 % DL-Met	0.380	0.340
55 % l-Lys H_2_SO_4_	0.670	0.500
98.5 % l-Thr	0.150	0.120
50 % choline	0.100	0.100
NaHCO_3_	0.150	0.150
Vitamin premix^1^	0.150	0.150
Mineral premix^2^	0.150	0.150
Total	100	100

Calculated energy and nutrient contents^3^		
AME_n_, kcal/kg	3,013	3,100
CP, %	22.3	19.5
Digestible Lys, %	1.25	1.08
Digestible Met + Cys, %	0.960	0.860
Digestible Met, %	0.530	0.480
Digestible Thr, %	0.835	0.720
Digestible Trp, %	0.200	0.170
Total calcium, %	0.850	0.650
Available phosphorus, %	0.460	0.360

Analyzed energy and nutrient compositions of *Abelmoschus manihot* L. flower powder		
GE, kcal/kg	3,115	
DM, %	93.3	
Crude ash, %	7.41	
CP, %	12.2	
Ether extract, %	1.65	
Calcium, %	0.660	
Phosphorus, %	0.480	
Arg, %	0.470	
His, %	0.220	
Ile, %	0.430	
Leu, %	0.750	
Lys, %	0.590	
Met, %	0.230	
Phe, %	0.440	
Thr, %	0.470	
Trp, %	0.140	
Val, %	0.540	
Ala, %	0.700	
Asp, %	0.990	
Cys, %	0.180	
Glu, %	1.71	
Gly, %	0.620	
Pro, %	0.500	
Ser, %	0.560	
Tyr, %	0.290	
Saturated fatty acid, g/100 g		
Caprylic acid, C8:0	0.080	
Capric acid, C10:0	0.070	
Lauric acid, C12:0	0.180	
Myristic acid, C14:0	0.760	
Pentadecanoic acid, C15:0	0.280	
Palmitic acid, C16:0	20.3	
Margaric acid, C17:0	0.210	
Stearic acid, C18:0	2.52	
Arachidic acid, C20:0	0.950	
Unsaturated fatty acid, g/100 g		
Pentadecanoic acid, C15:1	0.120	
Palmitoleic acid, C16:1	1.01	
Magaoleic acid, C17:1	0.180	
Oleic acid, C18:1 ω−9	23.2	
Linoleic acid, C18:2 ω−6	27.7	
γ -Linolenic acid, C18:3 ω−6	1.46	
Linolenic acid, C18:3 ω−3	19.2	
Eicosenoic acid, C20:1 ω−9	1.90	

Abbreviations: MCP = monocalcium phosphate.

1 Provided per kilogram of the complete diet vitamin A (from vitamin A acetate), 12,000 IU; vitamin D_3_, 4,000 IU; vitamin E (from DL-α-tocopheryl acetate), 80 IU; vitamin K_3_, 4 mg; vitamin B_1_, 4 mg; vitamin B_2_, 10 mg; vitamin B_6_, 6 mg; vitamin B_12_, 20 μg; calcium pantothenate, 20 mg; folic acid, 2 mg; biotin, 200 μg; niacin, 60 mg.

2 Provided per kilogram of the complete diet: Zn (as ZnO), 85 mg; Mn (as MnSO_2_·H_2_O), 90 mg; Cu (as CuSO_4_·5H_2_O), 15 mg; Co (as CoCO_3_), 25 μg; I (as Ca (IO_3_)_2_·H_2_O), 1.5 mg; Se (as Na_2_SeO_3_), 25 μg.

3 Calculated values from the Arbor Acres broiler nutrition specifications (Aviagen, 2022).

### Animals, diets, and experimental design

The protocol for this experiment was reviewed and approved by the Institutional Animal Care and Use Committee at Chungbuk National University (CBNUA-2268-24-01). In this study, 144 1-d-old mixed-sex Arbor Acres broiler chicks were obtained from local commercial hatchery (Dongsan Hatchery, Cheonan, Republic of Korea). The chicks were allotted to individual cages (four-layer cage; 52 × 61 × 42 cm = width × length × height for each cage) ensuring a similar average BW of 35.0 ± 0.4 g among cages. All birds were allotted to 1 of 3 treatments with 6 replicates per treatment in a completely randomized design. Each replicate consisted of 8 chicks. The basal diet was formulated to meet or exceed according to the recommended nutrition specifications for broiler chickens in the Arbor Acres Broiler Nutrition Specifications (Aviagen, 2022). The detailed composition and nutrient concentrations of the basal diet are provided in Table 1. Two additional experimental diets were prepared by partially replacing corn with AMP at inclusion levels of 0.5 or 1.0 g/kg, while maintaining the overall nutrient composition of basal diet. All diets were provided in mash form. All diets and water were fed *ad libitum* for 35 d. Diets were supplied following a two-phase feeding program, comprising a grower phase (1 - 21 d) and a finisher phase (22 - 35 d). The experimental temperature was set at 30°C for the first week and was gradually lowered to 24°C by the end of the trial. The lighting program was maintained under constant lighting conditions during the experiment. The BW gain (**BWG**) and feed intake (**FI**) were measured at the conclusion of the experiment. Feed efficiency (**FE**) was calculated by dividing BWG with FI.

### Sample collection

At the conclusion of the experiment (35 d of age), one male broiler chicken per replicate with a BW close to the replicate mean was euthanized by CO_2_ asphyxiation. Subsequently, the bird was dissected, and images of the liver were captured to assess liver hemorrhages and to assign a fatty liver score. Following dissection, the liver, spleen, thymus, and bursa of Fabricius were excised and weighed to determine relative organ weight as a percentage of BW. Liver samples were immediately placed in a 1.7-mL microtube and stored at −80°C for subsequent analysis of malondialdehyde (**MDA**) and total antioxidant capacity (**TAC**).

### Liver characteristics

Liver hemorrhages and fatty liver score were assessed from 6 male birds per treatment group by five investigators using visual inspection. The color of the raw liver was rapidly evaluated using a colorimeter to determine CIE L*, a*, and b* values. Hemorrhage scoring ranged from 0 to 5, where 0 denoted absence of hemorrhages; 1, 10 indicated subcapsular petechial or ecchymotic hemorrhages; 2, more than 10 subcapsular petechial or ecchymotic hemorrhages; and 3-5, represented extensive hemorrhages with rupture of Glission’s capsule as specified by Rozenboim et al. (2016). Fatty liver score ranged from 1 to 5 based on liver coloration, with 1 representing normal liver coloration, and 2 to 5 denoting shades from dark red to light yellowish red.

### Antioxidant capacity

Oxidative stress indicators, specifically MDA and TAC levels in the liver, were determined using commercially available kits: the OxiSelect^TM^ TBARS Assay Kit (STA-330, Cell Biolabs, San Diego, CA) for MDA and the EZ-Total Antioxidant Capacity Assay Kit (DG-TAC200, DoGenBio, Seoul, Republic of Korea) for TAC. Concentrations of MDA and TAC determination adhered strictly to the manufacturer’s instructions. Protein concentrations in liver samples were analyzed with a bicinchoninic acid protein assay kit (Thermo Scientific, Rockford, IL), a critical parameter in calculating antioxidant capacity.

### Statistical analysis

All data were analyzed using a completely randomized design with the PROC MIXED procedure in SAS (SAS Institute Inc., Cary, NC). Replicates served as the experimental unit for all data analyses. Outlier detection was conducted utilizing the UNIVARIATE procedure of SAS, confirming that no outliers were present. Treatment means were estimated using the LSMEANS procedure, and the PDIFF option in SAS was employed for mean separation when significant differences were observed. Orthogonal polynomial contrast tests were applied to assess both linear and quadratic effects of increasing inclusion levels of AMP in diets. Statistical significance and tendency were defined at *P* < 0.05 and 0.05 ≤ *P* < 0.10, respectively.

## Results and discussion

### Growth performance

The flower of *Abelmoschus manihot* L., similar to other medicinal plant flowers, contains a diverse array of bioactive compounds, including flavonoids, terpenoids, and steroids which may improve intestinal health and overall productivity in broiler chickens (Windisch et al., 2008). Specifically, these phytochemicals in the small intestine are expected to enhance nutrient digestibility and promote the proliferation of beneficial gut microbiota, which contributes to increased BWG and FE in broiler chickens. In general, improved intestinal function is vital for restricting the entry of pathoenhanceens and harmful lipopolysaccharides into the bloodstream, thereby reducing systemic inflammation. Nonetheless, in the current experiment, growth performance, including BW, BWG, FI, and FE, was not affected by dietary AMP supplementation in broiler chickens (Table 2). This unexpected outcome may partially result from variations in the composition and bioavailability of bioactive compounds in plant materials. Firstly, factors such as plant maturity at harvest, plant part utilization, geographical origin, environmental influences, and processing methods generally influence the levels and profiles of bioactive compounds. Secondly, degradation of some bioactive compounds may occur in the upper gastrointestinal tract before they reach their target sites in the intestine, diminishing their effectiveness. In particular, dietary supplementation of dry plant powder requires specific microbial enzymes to release phytobiotics from the plant matrix. However, this process may be constrained by insufficient enzyme availability in the intestine. These factors highlight the complexity in assessing the effects of PFAs, making it challenging to specify the exact mode of action for each bioactive compound, since they are present as combinations in plants. Additionally, it is likely that the optimal rearing conditions and the absence of environmental or pathogenic pressures in the current study may have concealed potential improvements from AMP supplementation on growth performance in broiler chickens. This occurrence is frequently described as a “masking effect”, where the lack of environmental or infectious stress prevents functional compounds from demonstrating their biological roles. Under such non-stressful conditions, birds may already achieve optimal immune and antioxidant capacities, thus lessening the impact of further physiological interventions (Gabai et al., 2020). Prior research supports that the efficacy of phytogenic feed additives in promoting growth tends to be more evident under stress-inducing environments such as heat stress, oxidative imbalance, or pathogen threats (Windisch et al., 2008). Taken together, these results imply that the antioxidant, anti-inflammatory, and immunomodulatory properties of AMP may not translate into observable benefits for the growth performance of broiler chickens.

**Table 2:** Effect of dietary supplementation of *Abelmoschus manihot* L. flower powder on growth performance, relative organ weight, and liver status in broiler chickens.^1^.

		Inclusion levels of AMP in diets, %				*P-*value^2^		
Items		0	0.5	1.0	SEM	T	L	Q
Growth performance								
BW, g		1,678	1,618	1,678	43.0	0.484	0.998	0.237
BWG, g		1,643	1,583	1,643	43.0	0.485	0.997	0.238
FI, g		2,389	2,368	2,455	69.2	0.644	0.494	0.507
FE, g/kg		689	669	669	10.3	0.268	0.182	0.392
Relative organ weight								
Liver, %		2.47	2.42	2.23	0.081	0.119	0.054	0.476
Spleen, %		0.124^a^	0.120^a^	0.080^b^	0.009	0.004	0.003	0.108
Kidney, %		0.579	0.614	0.607	0.030	0.687	0.519	0.551
bursa of Fabricius, %		0.247	0.231	0.264	0.017	0.592	0.495	0.452
Thymus, %		0.337	0.309	0.331	0.038	0.866	0.908	0.607
Liver characteristics								
Liver color	L*	22.5	21.9	22.9	0.600	0.465	0.644	0.256
	a*	15.9	14.6	15.2	0.734	0.473	0.501	0.312
	b*	3.30	2.58	3.37	0.386	0.310	0.904	0.133
Liver hemorrhagic score		0.467	0.367	0.467	0.088	0.659	1.000	0.369
Fatty liver score		1.23^a^	1.03^b^	1.10^b^	0.038	0.006	0.024	0.011
Liver antioxidant capacity								
MDA, μM/mg protein		3.32	2.49	2.46	0.428	0.281	0.159	0.419
TAC, nmol/mg protein		0.491	0.508	0.656	0.057	0.085	0.049	0.329

Abbreviations: AMP = *Abelmoschus manihot* L. flower powder; BWG = BW gain; FI = feed intake; FE = feed efficiency (BWG:FI); L* = lightness; a* = redness; b* = yellowness (liver color); MDA = malondialdehyde; TAC = total antioxidant capacity.

a,b Means within a variable with no common superscript differ significantly (*P* < 0.05).

1 Data are least squares means of 6 observations per treatment.

2 T = overall effects of treatments; *L* = linear effects of increasing inclusion levels of *Abelmoschus manihot* L. flower powder in diets; *Q* = quadratic effects of increasing levels of *Abelmoschus manihot* L. flower powder in diets.

### Relative organ weight

A trend toward decreased (linear, *P* = 0.054) relative liver weight was noted with increasing inclusion levels of AMP in diets (Table 2). Birds fed diets containing 1.0 % AMP had less (*P* < 0.05) relative spleen weight than those fed diets containing 0 and 0.5 % AMP. Moreover, there was a decrease (linear, *P* < 0.05) in relative spleen weight as inclusion levels of AMP in diets increased. However, increasing inclusion levels of AMP in diets did not affect relative weights of kidney, bursa of Fabricius, and thymus. This effect may be linked to the presence of bioactive compounds in AMP, such as flavonoids and polysaccharides, which have been recognized for their roles in organ development and immune system modulation. These compounds likely promote hepatic metabolic function by mitigating lipid accumulation and minimizing oxidative damage in liver tissues. Functional PFAs have been shown to reduce hepatic lipid buildup by modulating key metabolic pathways, including peroxisome proliferator-activated receptor α and adenosine monophosphate-activated protein kinase, both of which regulate β-oxidation and lipogenesis (Flees et al., 2021). Furthermore, the antioxidant capabilities of flavonoids help maintain hepatocyte health by neutralizing reactive oxygen species and protecting against oxidative stress-induced liver enlargement (Chodkowska et al., 2022). Moreover, the antioxidant effects may suppress lymphocyte proliferation in the spleen, leading to a reduction in spleen weight (Flees et al., 2021). Consequently, the decreases in relative liver and spleen weights may be attributed to the actions of AMP-derived bioactive compounds, which have been reported to improve metabolic processes and protect tissues from damage. The liver and spleen are immunologically reactive organs that can exhibit rapid responses to a small amount of dietary bioactive compounds. Therefore, assessing organ weight in isolation may not adequately reveal all underlying physiological mechanisms. It is important to interpret organ weight changes together with additional physiological and histopathological parameters, since shifts in organ mass may represent adaptive physiological processes rather than indicate pathological lesions.

### Liver characteristics

As shown in Table 2, the fatty liver scores for birds fed diets containing 0.5 and 1.0 % AMP were less (*P* < 0.05) than for those fed the basal diet. In addition, fatty liver score was decreased (linear and quadratic, *P* < 0.05) with increasing inclusion levels of AMP in diets. However, liver color and liver hemorrhagic score were not affected by increasing inclusion levels of AMP in diets. The liver, the largest metabolic organ in poultry, is known for vital and complex physiological roles, such as nutrient metabolism, excretion, detoxification, immune regulation, and lipid processing. Consequently, assessment of liver attributes, including liver color, fatty liver score, and hemorrhagic score, may serve as an effective means of evaluating hepatic health and physiological responses to nutritional interventions. The liver oxidizes fatty acids obtained from the diet to generate the energy necessary for hepatic metabolism and transforms excess dietary fatty acids into triglycerides (**TG**), which are subsequently distributed to peripheral tissues. Nevertheless, excessive TG production can lead to fat accumulates within hepatocytes, thereby impairing liver function. In this study, fatty liver score decreased in a linear manner as dietary AMP inclusion levels increased. To date, there are no published studies examining the effects of dietary supplementation of *Abelmoschus manihot* L. on liver characteristics in broiler chickens. As a result, direct comparison of our findings with previous poultry data was not possible. However, similar improvements in liver characteristics have been reported in studies utilizing other PFAs. While the specific mechanism by which PFAs influence hepatic lipid metabolism remain to be fully elucidated, their bioactive constituents, including flavonoids and polyphenols, are recognized for their antioxidant and anti-inflammatory effects, which likely help decrease hepatic fat accumulation in poultry (Flees et al., 2021; Chodkowska et al., 2022). Additionally, AMP has been identified to contain quercetin, a naturally sourced polyphenolic molecule, which reduces hepatic fat accumulation by alleviating oxidative stress. It suppresses the expression of pro-inflammatory cytokines, including tumor necrosis factor alpha, interleukin-6, and cyclooxygenase-2, and inhibits nuclear factor-κB by modulating the toll-like receptor 4 signaling pathway (Zou et al., 2015). These results further indicate that polyphenolic compounds in PFAs may contribute to reducing hepatic fat deposition in poultry.

### Liver antioxidant capacity

Supplementation with 1.0 % AMP in diets tended to increase (*P* = 0.085) TAC levels compared to those fed basal diet (Table 2). In addition, increasing inclusion levels of AMP in diets increased (linear, *P* < 0.05) TAC levels in the liver. However, increasing inclusion levels of AMP in diets did not significantly affect MDA levels in the liver. The linear increase in TAC suggests that dietary AMP supplementation enhanced the hepatic antioxidant system. This enhancement may have prevented the accumulation of oxidative damage, thereby maintaining MDA levels stable while antioxidant capacity improved. Antioxidant activity generally serves as a crucial biomarker that reflects the physiological, pathological, and nutritional status of animals. The exact mechanism underlying this observation remains unclear; however, it is hypothesized that the elevated TAC may be attributed to the presence of abundant bioactive compounds within AMP. Flavonoids represent the principal active constituents of *Abelmoschus manihot* L., accounting for approximately 6 % of the dry weight of the flower, which exceeds that found in most conventional plants. Among these, hyperin, a flavonoid glycoside present in AMP, has been reported to alleviate oxidative stress by activating the transcription factor NF-E2-related factor 2 (**Nrf2**) signaling pathway (Song et al., 2025). Similarly, Liu et al. (2021) observed that *Abelmoschus manihot* L. flower extract significantly upregulated the expression of Nrf2 and antioxidant enzymes in H_2_O_2_-induced HepG2 cells, diminished MDA levels and enhanced glutathione peroxidase activity. Nrf2 is recognized as a key regulator in cellular defense mechanisms against oxidative stress and apoptosis. Additionally, *Abelmoschus manihot* L. is known to contain other antioxidant compounds such as tocopherols, carotenoids, and phenolics, which have been confirmed to effectively neutralize reactive oxygen species, improve antioxidant capacity, and support cellular redox balance (Kwon et al., 2022). However, this study is limited in its capacity to ascertain whether the observed antioxidant effect is due to an individual bioactive compound (e.g., hyperin) or results from synergistic effects among various bioactive compounds, including those not specifically identified in AMP, such as tocopherols and carotenoids. In summary, the results suggest that dietary AMP supplementation at 1.0 % improved the hepatic antioxidant capacity and attenuated fat accumulation as a functional feed additive without negative impacts on growth performance in broiler chickens. These findings may provide insights into the future application of AMP as functional feed additive in broiler chickens and offer a potential nutritional strategy for improving liver function in modern poultry production. However, further studies are warranted to elucidate the underlying mechanisms whereby individual bioactive compounds of AMP influence physiological responses in broiler chickens.

## Acknowledgements

This research was supported by “Regional Innovation Strategy (RIS)” through the National Research Foundation of Korea(NRF) funded by the Ministry of Education(MOE) (2021RIS-001). This work was supported by the National Research Foundation of Korea (NRF) grant funded by the Korea government (MSIT) (No. RS-2023-00210634).

## CRediT authorship contribution statement

**Gyu Lim Yeom:** Writing – review & editing, Writing – original draft, Software, Methodology, Investigation, Formal analysis, Data curation. **Ha Neul Lee:** Writing – review & editing, Methodology, Investigation, Formal analysis. **Yeong Bin Kim:** Writing – review & editing, Methodology, Investigation, Formal analysis. **Ju Yeong Park:** Writing – review & editing, Methodology, Investigation, Formal analysis. **Geun Yong Park:** Investigation, Formal analysis. **Ji Won Shin:** Investigation, Formal analysis. **Yang-il Choi:** Writing – review & editing, Methodology, Data curation, Conceptualization. **Jungseok Choi:** Writing – review & editing, Methodology, Data curation, Conceptualization. **Jong Hyuk Kim:** Writing – review & editing, Writing – original draft, Validation, Supervision, Project administration, Funding acquisition, Conceptualization.

## Disclosures

The authors declare that they have no known competing financial interests or personal relationships that could have appeared to influence the work reported in the present study.
